# Elovl6 is a negative clinical predictor for liver cancer and knockdown of Elovl6 reduces murine liver cancer progression

**DOI:** 10.1038/s41598-018-24633-3

**Published:** 2018-04-26

**Authors:** Yu-Chu Su, Yin-Hsun Feng, Hung-Tsung Wu, Yao-Shen Huang, Chao-Ling Tung, Pensee Wu, Chih-Jen Chang, Ai-Li Shiau, Chao-Liang Wu

**Affiliations:** 1 Department of Otolaryngology, National Cheng Kung University Hospital, College of Medicine, National Cheng Kung University, Tainan, Taiwan; 2 Department of Biochemistry and Molecular Biology, College of Medicine, National Cheng Kung University, Tainan, Taiwan; 3 Division of Hematology and Oncology, Department of Internal Medicine, Chi-Mei Medical Center, Yong Kang, Tainan, Taiwan; 4 Department of Nursing, Chung Hwa University of Medical Technology, Tainan, Taiwan; 5 Department of Family Medicine, College of Medicine, National Cheng Kung University, Tainan, Taiwan; 6 Institute for Science & Technology in Medicine, Keele University, Keele, United Kingdom; 7 Department of Microbiology and Immunology, College of Medicine, National Cheng Kung University, Tainan, Taiwan

## Abstract

The elongation of long-chain fatty acids family member 6 (Elovl6) is a key enzyme in lipogenesis that catalyzes the elongation of saturated and monounsaturated fatty acids. Insulin resistance involves upregulation of Elovl6, which has been linked to obesity-related malignancies, including hepatocellular carcinoma (HCC). However, the role of Elovl6 in cancer progression remains unknown. In this study, we analyzed the expression of Elovl6 in 61 clinical HCC specimens. Patients with Elovl6 high-expressing tumors were associated with shorter disease-free survival and overall survival compared to those with Elovl6 low-expressing tumors. Knockdown of Elovl6 in HCC cells reduced cell proliferation and Akt activation, as well as sensitivity to fatty acids. Inhibition of Elovl6 reduced tumor growth and prolonged survival in mice bearing tumors. Taken together, our results indicate that Elovl6 enhances oncogenic activity in liver cancer and is associated with poor prognosis in patients with HCC. Elovl6 may be a therapeutic target for HCC; thus, further studies to confirm this strategy are warranted.

## Introduction

Obesity has become one of the important public health problems worldwide, and its prevalence has significantly increased in the last few decades. Obesity associated with metabolic syndrome often causes multiple morbidities, including insulin resistance, type 2 diabetes mellitus, and non-alcoholic fatty liver disease. The pathological spectrum of non-alcoholic fatty liver disease extends from simple steatosis to the more severe non-alcoholic steatohepatitis (NASH), leading to liver cirrhosis and eventually to hepatocellular carcinoma (HCC)^1^. The underlying mechanisms of hepatocarcinogenesis related to obesity and insulin resistance have been extensively studied.

Lipogenesis is involved in the energy storage system. The elongation of long-chain fatty acids family member 6 (Elovl6) is part of a highly conserved family of endoplasmic reticulum enzymes involved in the formation of long-chain fatty acids. Elovl6 specifically catalyzes the elongation of saturated and monounsaturated fatty acids with 12, 14, and 16 carbons. Dietary polyunsaturated fatty acids cause profound suppression of Elovl6 expression^2^. Microarray analysis of sterol regulatory element-binding protein-1 (SREBP-1) transgenic mice has revealed that Elovl6 is a direct target of SREBP-1 and is regulated directly and primarily by SREBP-1c^2,3^. Therefore, Elovl6 has been shown to increase insulin resistance in fatty livers, even with concurrent obesity in mice^4^. Since Elovl6 expression is positively correlated with the severity of hepatosteatosis and liver injury in NASH, deletion of Elovl6 reduces palmitate-induced activation of the NOD-like receptor family pyrin domain-containing 3 (NLRP3) inflammasome, suggesting that Elovl6 modulates the progression of NASH^5^. In addition, a 10-year follow-up study of a nationwide population-based cohort demonstrated that the population attributable fraction of site-specific cancer risks among patients with type 2 diabetes was highest for liver cancer followed by pancreatic and kidney cancers^6^. In the present study, we investigated the effect of Elovl6 on tumor growth *in vitro* and *in vivo* and analyzed the correlation between Elovl6 and clinical features to better understand the role of Elovl6 in HCC.

## Results

### Upregulation of Elovl6 expression is detected in tumor specimens of HCC patients with poor outcome

**Figure 1:**
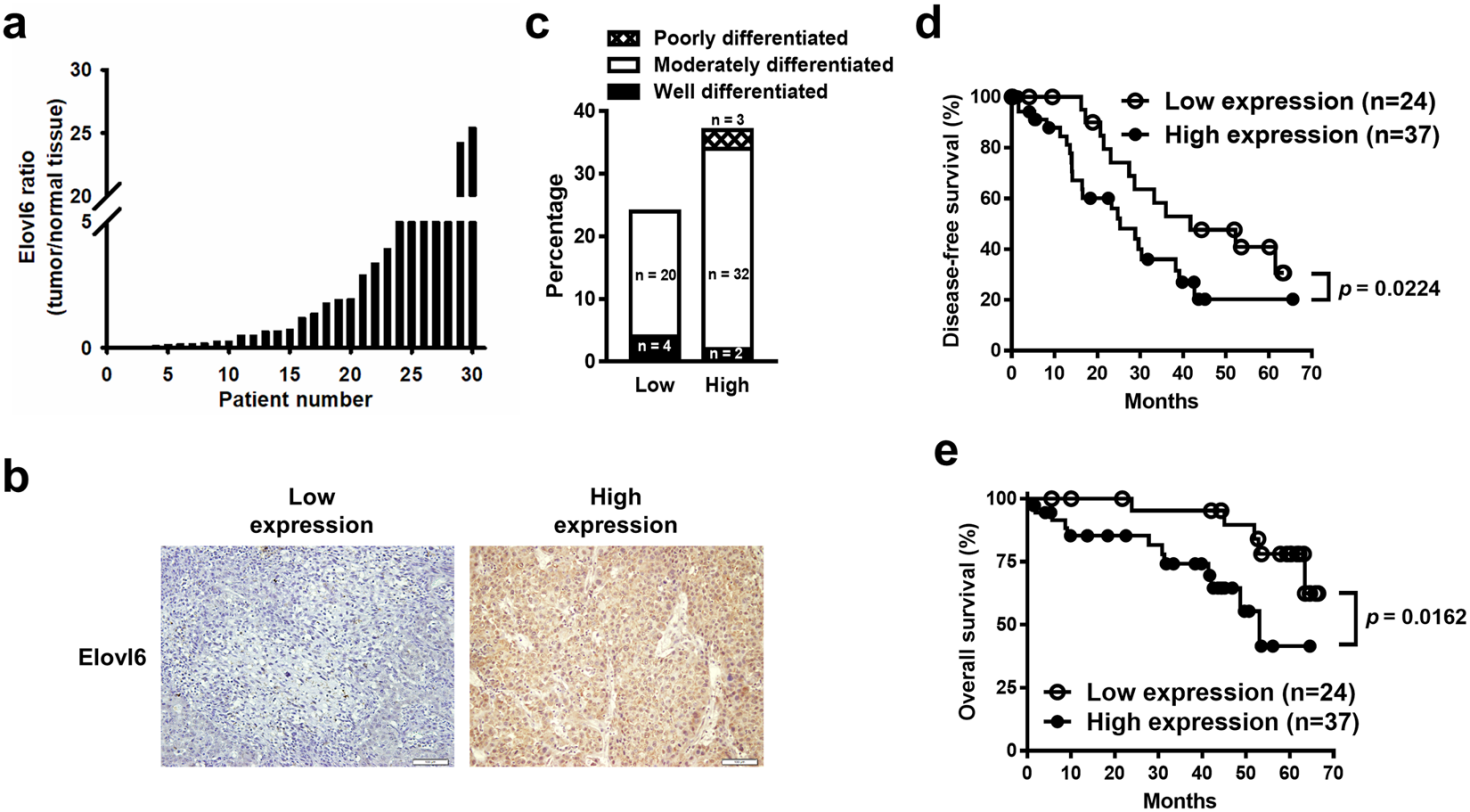
Expression of Elovl6 in human hepatocellular carcinoma. (**a**) RNA transcripts of Elovl6 expression in thirty hepatocellular carcinomas. (**b**) Immunostaining intensity of clinical samples with low or high expression of Elovl6. (**c**) The correlation of expression of Elovl6 and differentiation in HCC samples. Disease-free (**d**) and overall (**e**) survival in HCC patients with high or low expression of Elovl6. Statistical differences were analyzed using the log-rank test.

We first analyzed the mRNA expression levels of Elovl6 in 30 patients with HCC. As shown in Fig. 1a, the expression levels of Elovl6 varied in different tumors but were increased in 50% of the tumors examined compared with their adjacent normal tissue. To address the impact of Elovl6 on the outcome of human HCC, 61 tumor specimens from resected HCC were stained for Elovl6. The intensity of immunostaining was classified as low expression (H-scores between 0 and 139) and high expression (H-scores ≥ 140; Fig. 1b). A univariate analysis showed that expression of Elovl6 had no significant association with stage, age, gender, or differentiation. The expression of Elovl6 did not correlate significantly with diabetes mellitus, body mass index (BMI), hepatitis B, or hepatitis C (Table 1). Interestingly, patients with higher BMIs (>25) tended to have high Elovl6-expressing HCC, but this did not reach statistical significance (*p* = 0.581). None of the Elovl6 low-expressing tumors were in the poor differentiation group (Fig. 1c). Furthermore, patients with high Elovl6 expression had significantly poorer prognoses, including shorter disease-free survival (Fig. 1d) and reduced overall survival (Fig. 1e), compared with those with low Elovl6 expression. Taken together, these data suggest that high expression of Elovl6 may be associated with cancer progression.

**Table 1:** Clinical characteristics of HCC tissues with high or low expression of Elovl6.

Elovl6	High expression n = 37	Low expression n = 24	p
Age median (range)	64 (47–79)	61 (23–78)	0.623
Gender (male/female)	21/16 (57%/43%)	19/5 (79%/21%)	0.128
BMI median (range) BMI >25	24.6 (20.4–32.6) 13 (35%)	22.6 (15.5–31.6) 6 (25%)	0.154 0.581
Diabetes mellitus	6 (16%)	5 (21%)	0.738
Hepatitis B	23 (62%)	12 (50%)	0.501
Hepatitis C	16 (43%)	10 (41%)	0.886

Test for age and BMI(median): Mann-Whitney Rank Sum Test.

Test for Gender, BMI(percentage), Diabetes mellitus, Hepatitis B, Hepatitis C: Chi-square test.

### Knockdown of Elovl6 expression changes cell morphology and sensitivity to fatty acids in HCC cells

**Figure 2:**
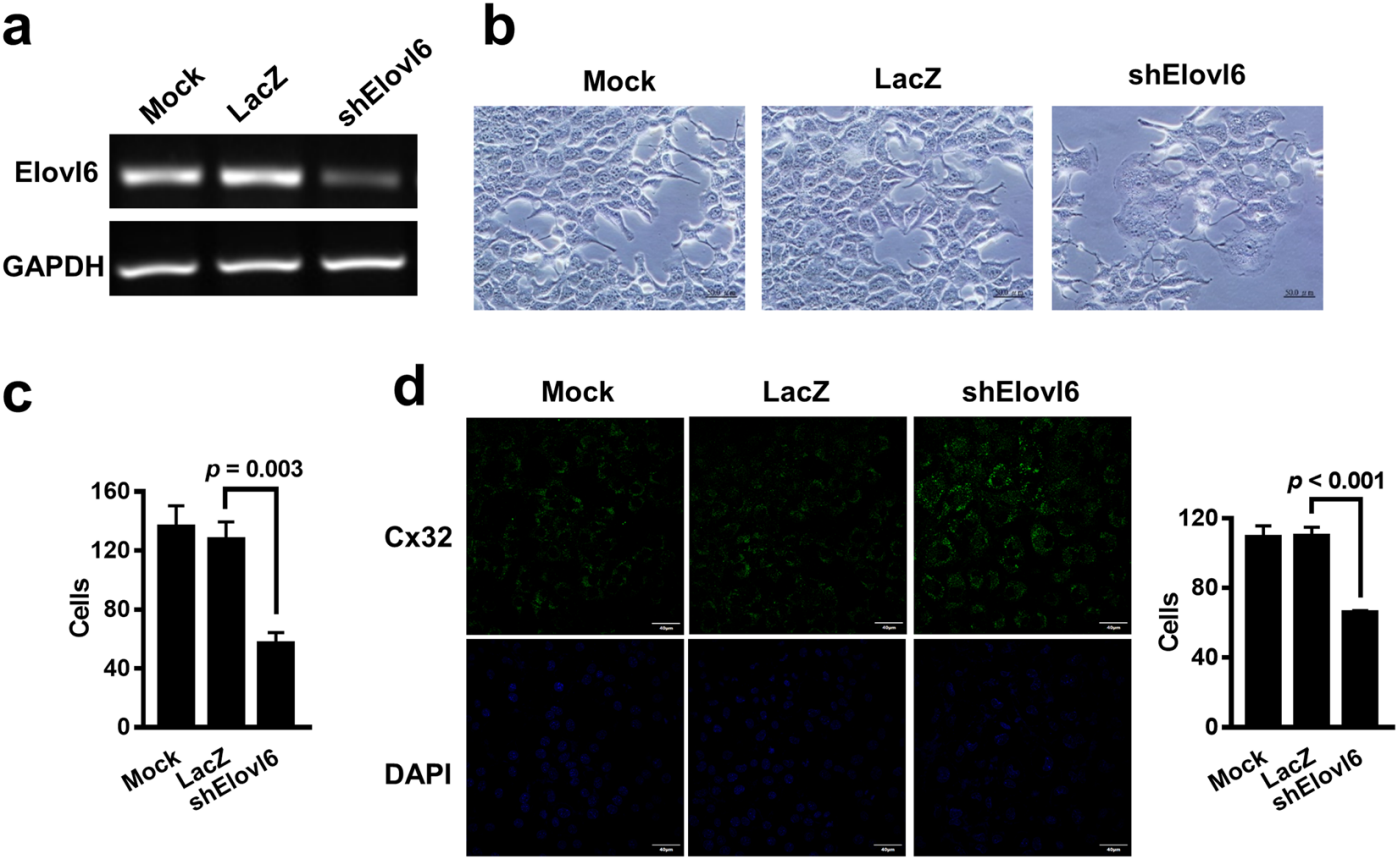
Deletion of Elovl6 expression alters cell morphology and cell-cell junction. (**a**) ML-1 cells were infected with Ad.shElovl6 or Ad.LacZ. Expression of Elovl6 was examined by RT-PCR. (**b**,**c**) Cell morphology was detected after 72 h of infection (**b**), and the cell number was calculated (**c**). Scale bar = 50 μm; original magnification, 400× . (**d**) Expression of gap junction protein Cx32 was examined in the Elolv6 knockdown ML-1 cells using immunofluorescence, and the number of cells in each group was counted. Scale bar = 40 μm; original magnification, 400× .

To investigate the role of Elovl6 in HCC, adenoviral vectors carrying Elovl6 shRNA were used to silence its expression in HCC cells. Knockdown of Elovl6 was verified with RT-PCR analysis in mouse ML-1 liver cancer cells infected with Ad.shElovl6 at 10 multiplicities of infection (MOI) (Fig. 2a). Elovl6 knockdown cells exhibited increased cell mass and tight gaps between cells (Fig. 2b). The number of Elovl6 knockdown cells was also decreased compared with the control cells (Fig. 2c). We further examined expression of the gap junction protein Cx32 in Elovl6 knockdown ML-1 cells. The immunofluorescence results showed that knockdown of Elovl6 increased expression of Cx32 (Fig. 2d). This implied that suppression of Elovl6 resulted in lower cell numbers and tighter cell-cell junctions.

**Figure 3:**
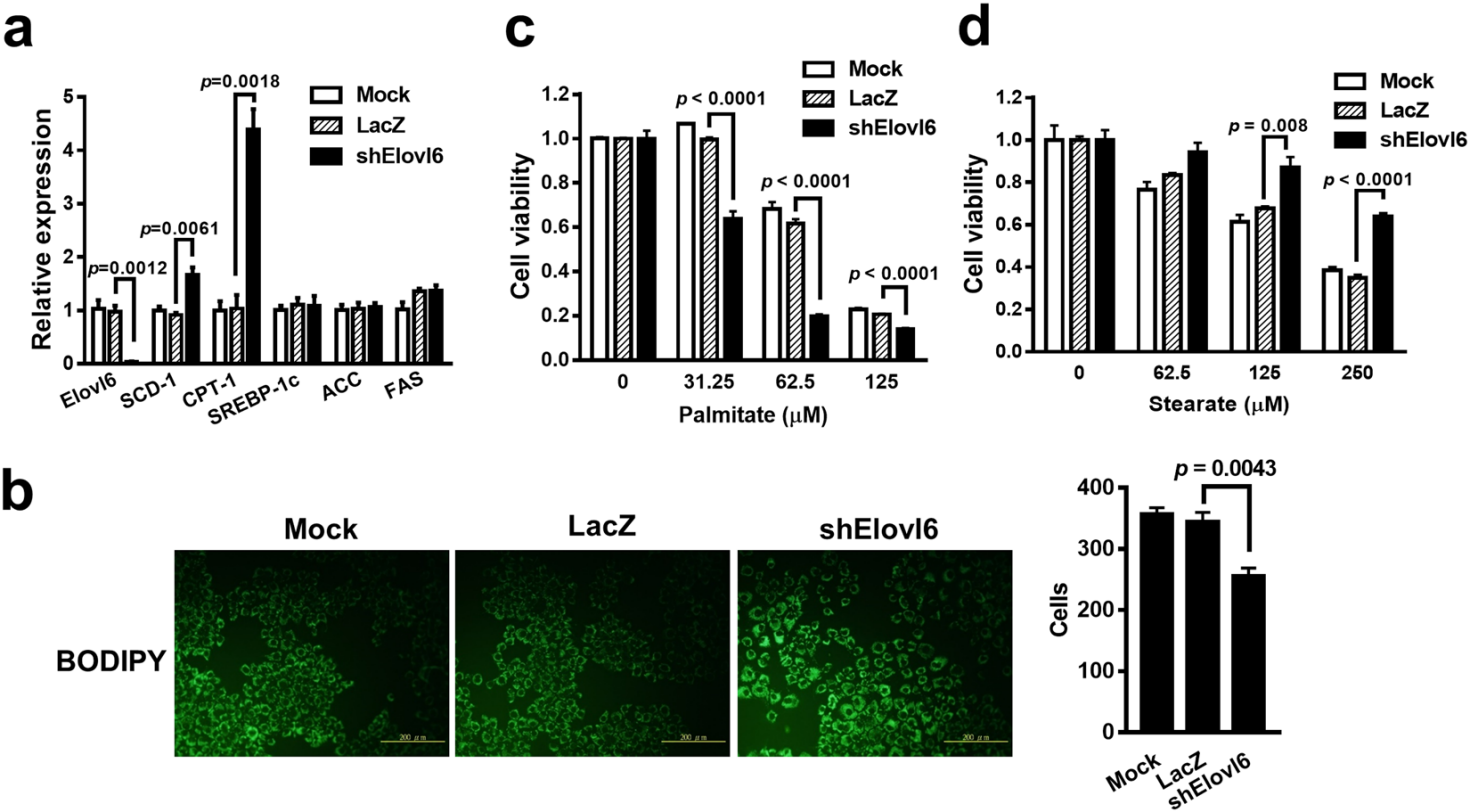
Elovl6 knockdown leads to lipid accumulation. (**a**) The lipid metabolism-related genes were analyzed using real-time RT-PCR after 48 h of infection. (**b**) The lipid accumulation in cells was detected using the BODIPY stain. Scale bar = 100 μm; original magnification, 200× . Cell numbers for the three groups were counted. (**c**,**d**) After 24 h of infection, the cells were treated with palmitate (**c**) or stearate (**d**) for 72 h. Cell viability was measured with the WST-8 assay. Data are mean ± S.E.M.

Given that Elovl6 plays a role in lipid metabolism^4^, we next examined the expression levels of lipid metabolism-related genes to investigate whether Elovl6 regulates lipid metabolism. The expression levels of the fatty acid desaturase SCD-1 and fatty acid oxidation gene CPT-1 were increased in Elovl6 knockdown cells, whereas those of SREBP-1c, ACC, and FAS were similar in both the Elovl6 knockdown and control cells (Fig. 3a). A BODIPY stain revealed that lipid accumulation was significantly elevated in the Elovl6 knockdown cells (Fig. 3b). Furthermore, reduction of Elovl6 expression affected cell sensitivity to fatty acids. The Elovl6 knockdown cells were more sensitive to palmitate-induced cell death (IC_50_ was reduced from 67 µM to 31 µM) and were insensitive to stearate treatment (IC_50_ was increased from 192 µM to 358 µM) (Fig. 3c,d). Taken together, inhibition of Elovl6 expression results in lipid accumulation and retards cell growth.

### Knockdown of Elovl6 expression causes cell cycle arrest and inhibits cell proliferation

**Figure 4:**
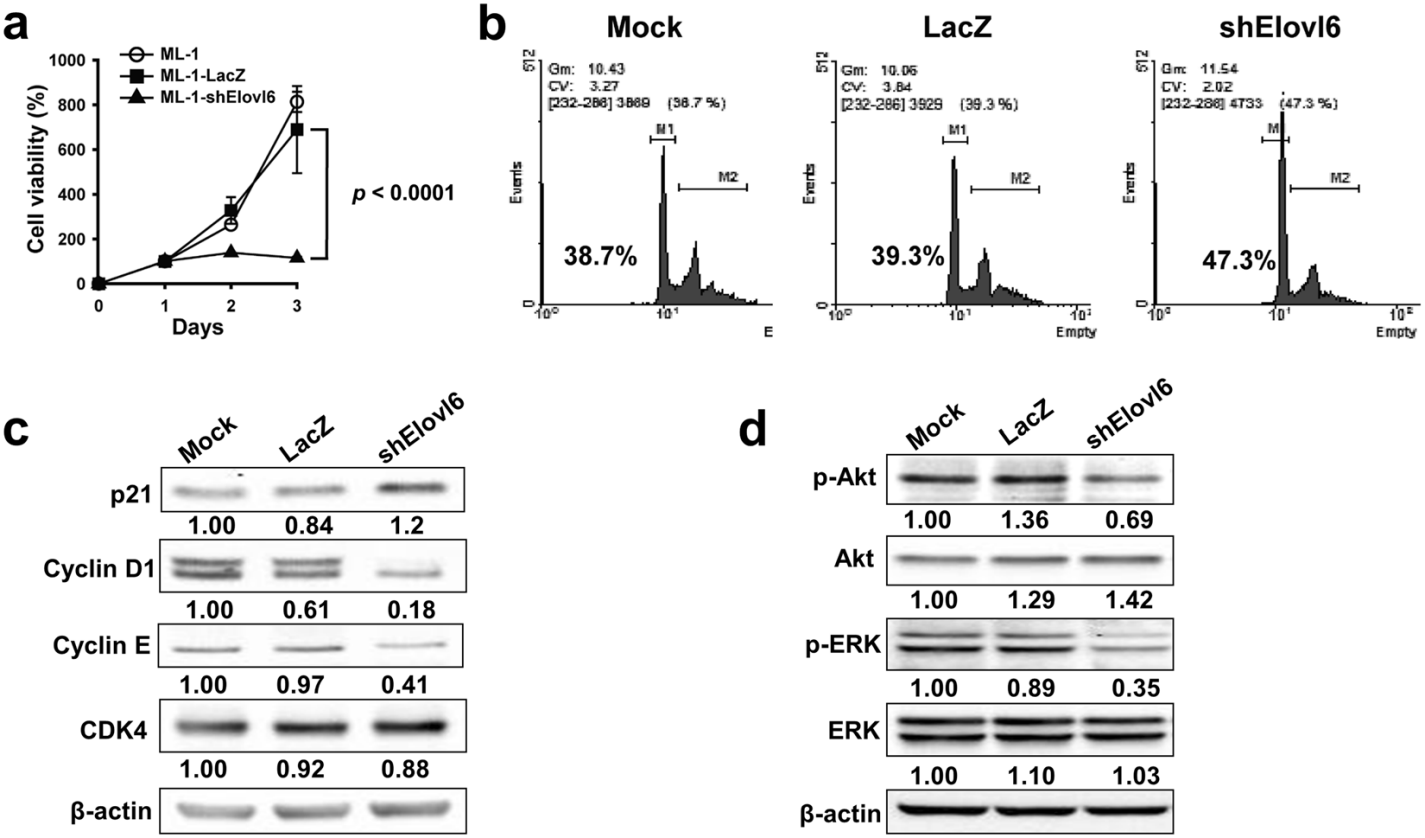
Inhibition of Elovl6 expression causes cell cycle arrest in ML-1 cells. (**a**) Cell growth was analyzed with the WST-8 assay at the indicated time. (**b**) The cell cycle analysis was quantified using flow cytometry. M1 = G1/S phase; M2 = G2/M phase. The values shown in the graphs are cell percentages in the G1/S phase. (**c**) Cell cycle-related proteins were determined by Western blot. (**d**) Phosphorylation of Akt and ERK were detected using Western blot. Data are mean ± S.E.M. The full-length blots are presented in Supplementary Fig. S3.

Since there were fewer Elovl6 knockdown ML-1 cells than control cells (Fig. 2c), we examined the rate of cell proliferation. The rate of cell growth was significantly reduced in the Elovl6 knockdown cells compared with the two control cells (Fig. 4a). We further investigated whether knockdown of Elovl6 results in cell death. No apoptotic cells were detectable in the Elolv6 knockdown ML-1 cells. (Supplementary Fig. S1). Furthermore, the cell cycle was analyzed using PI staining and flow cytometry. The percentage of the G1/S phase was increased in the Elovl6 knockdown cells compared with the control cells (Fig. 4b). In addition, knockdown of Elovl6 expression led to reduced expression of the cell cycle-related genes cyclin D1 and E, as well as increased expression of p21, but did not change the level of CDK4 expression (Fig. 4c). PI3K/Akt and MAPK pathways have been shown to be overactivated in liver cancers and to play a role in cell proliferation^7^. Figure 4d shows that phosphorylated Akt and ERK levels were significantly decreased in the Elolv6 knockdown cells. These results suggest that the inhibition of cell growth caused by Elovl6 knockdown may be mediated through Akt and MAPK pathways.

### Suppression of Elovl6 reduces tumor growth in mice bearing syngeneic HCC

**Figure 5:**
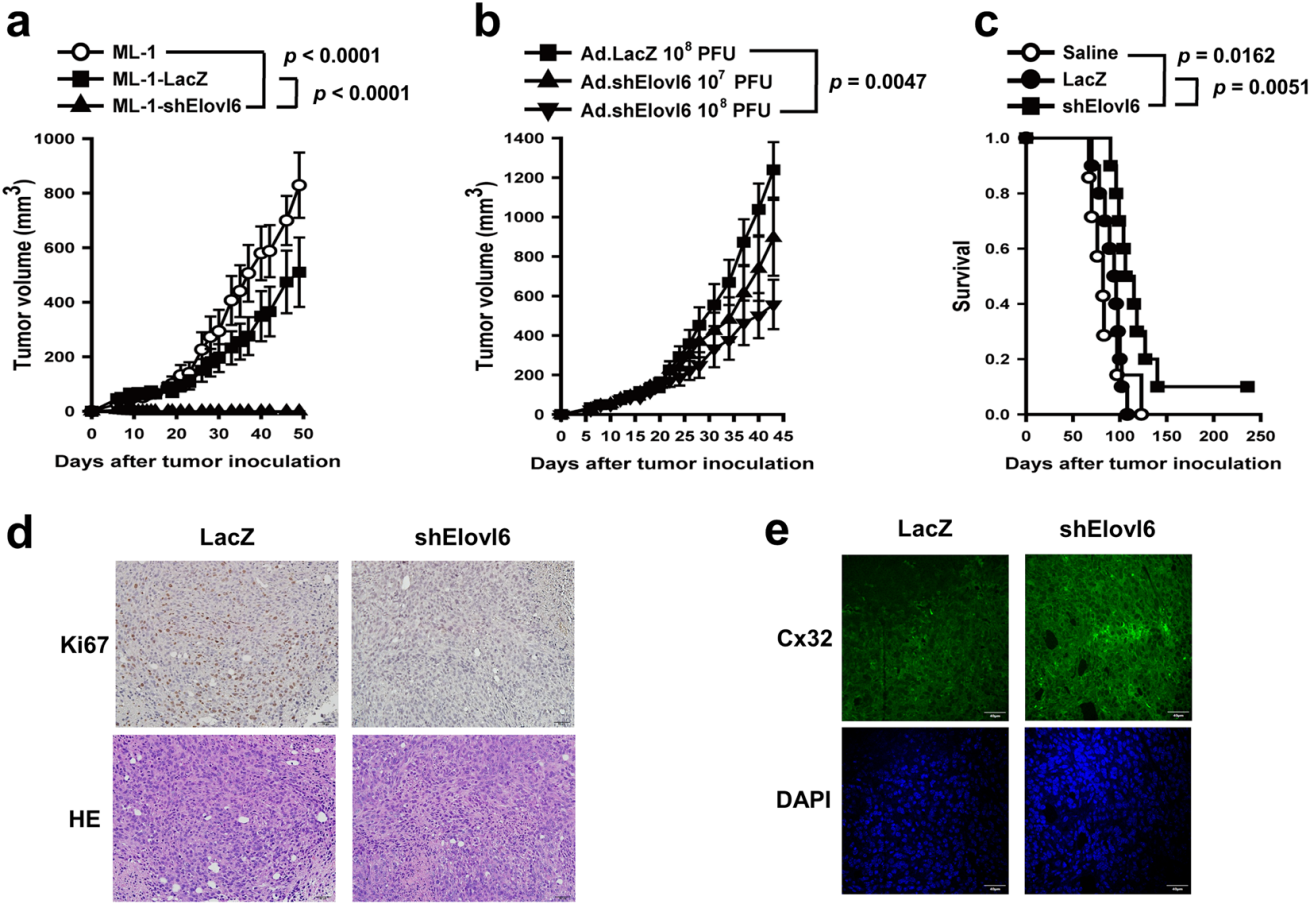
Knockdown of Elovl6 expression decreases tumor growth in mice. (**a**) Mice were inoculated subcutaneously with ML-1-shElovl6 or control cells. Tumor volumes were monitored twice a week. (**b**) Tumor-bearing mice were treated intratumorally with 10^7^ or 10^8^ PFU adenoviruses on days 10 and 16. (**c**) Kaplan-Meier survival curves for each group of mice. Statistical differences were analyzed with the log-rank test. (**d**,**e**) Ki67-positive cells (d) and gap junction protein Cx32 (e) were examined in the Elovl6-knockdown tumor samples on day 14. Scale bar = 50 μm (d) and 40 μm (e); original magnification, 200× (d) and 400× (e).

Given that Elovl6 expression caused lipid accumulation (Fig. 3b) and resulted in reduced cell growth (Fig. 4a), we further investigated whether suppression of Elovl6 expression inhibited tumor growth *in vivo*. First, BALB/c mice were inoculated subcutaneously with ML-1-shElovl6, ML-1-LacZ, or parental ML-1 cells, and their tumor growth was monitored. Whereas two control tumors grew progressively, Elovl6 knockdown tumors were diminished 10 days after tumor cell inoculation and did not recur during the 50-day observation period (Fig. 5a). To further study the role of Elovl6 in tumor growth, BALB/c mice were inoculated subcutaneously with parental ML-1 cells on day 0, followed by an intratumoral injection of either 10^7^ or 10^8^ PFUs of Ad.shElovl6 on days 10 and 16. Treatment with Ad.shElovl6 significantly suppressed tumor growth (Fig. 5b) and prolonged survival (Fig. 5c) in tumor-bearing mice, as compared to treatment with the control vector or saline. Moreover, Ki67-positive cells were decreased, but no apoptotic signals were detected in the Elovl6 knockdown group (Fig. 5d and Supplementary Fig. S2). Simultaneously, inhibition of Elovl6 led to increased Cx32 expression in the tumor samples (Fig. 5e). Collectively, knockdown of Elovl6 expression in HCC suppresses tumor growth and enhances survival in mice bearing syngeneic HCC.

## Discussion

Elovl6 is a key enzyme in the elongation of 16-carbon fatty acid to 18-carbon. A previous study showed that suppression of Elovl6 led to composition changes in fatty acids with increased 16-carbon and decreased 18-carbon^5^. Given that 16-carbon palmitate is toxic to cells^8^, unexpectedly, there was no apoptotic cell observed in both the Elovl6 knockdown cells and Elovl6 knockdown tumor samples. These results suggested that accumulation of 16-carbon fatty acids only resulted in arresting cell growth but not in cell death. Additionally, Elovl6 knockdown cells were more sensitive to palmitate-treatment but more tolerant to stearate-treatment. This implied that Elovl6 knockdown-induced fatty acid accumulation might not be sufficient to induce cell death or may not be able to detect cell death at the experiment time. Furthermore, expression of fatty acid desaturase SCD-1 and fatty acid oxidation gene CPT-1 were increased in the Elovl6 knockdown cells, suggesting that production of palmitoleate (C16:1) and fatty acid oxidation were promoted in the Elovl6 knockdown cells to reduce the toxicity of palmitate (C16:0).

Matsuzaka *et al*. showed that Elovl6 knockout restores insulin-induced Akt phosphorylation and thus ameliorates insulin resistance. Depletion of Elovl6 suppresses PKCε translocation to the cell membrane and inhibits PKCε function^4^. PKCε activation participates in the pathogenesis of lipid-induced insulin resistance through defecting insulin-stimulated IRS-2 tyrosine phosphorylation^9^. Elovl6 knockout increases Akt phosphorylation under insulin treatment in mice hepatocytes. However, Matsuzaka and his colleagues showed that Elolv6 might decrease Akt phosphorylation under normal conditions^4^. These findings were consistent with those of our study, where suppression of Elovl6 decreased Akt phosphorylation and caused cell cycle arrest. This suggests that insulin might be a key regulator of Elovl6 in the promotion or inhibition of Akt phosphorylation.

In recent years, metabolic syndromes have become a major issue in public health. Cancer metabolism has been identified as a novel cancer therapeutic strategy aside from conventional cytotoxic chemotherapy. In order to target the metabolism of tumors, many studies are focusing on the lipogenesis pathway. Upregulation of Elovl6 transcripts was detected in mice with hepatocytic deletion of Pten, which led to NASH and HCC later in life^10^. Although here, we show that silencing of Elovl6 expression changed cell morphology and sensitivity to fatty acids in liver cancer cells, the determination of the oncogenic role of Elovl6 remains challenging. Using the Gene Expression Omnibus dataset, decrease of Elovl6 mRNA expression was found in the majority of 247 HCC samples compared with the mean of 239 non-tumor liver tissues^11^. In the present study, 30 pairs of HCC sample and non-tumor liver tissues were analyzed for mRNA expression of Elovl6. 15 cases (50%) had Elovl6 ratios (tumor/non-tumor liver tissue) less than 1, which indicated that Elovl6 contributed to tumorigenesis only in a specific part of HCC. Since etiologies of liver carcinogenesis still include diverse causes, including alcohol, viruses, etc. An examination of Elovl6 in HCC cases with different etiologies is thus encouraged to define the contribution of Elovl6 in HCC.

Obesity is known to contribute to increased risk of NASH and HCC, mainly mediated by insulin resistance and adipokines^12^. Elovl6 has been extensively investigated in relation to insulin resistance; however, its role in HCC has seldom been studied. Phosphorylation of Akt is activated by insulin stimulation, and Elovl6 suppresses Akt phosphorylation in the presence of insulin in mouse hepatoma cells^4^. In the present study, we found that knockdown of Elovl6 expression caused cell cycle arrest and inhibited tumor cell proliferation through the inhibition of the Akt-mediated signaling pathway. All these findings suggest that Elovl6 is involved in oncogenic signaling. Thus, inhibition of Elovl6 may be a potential strategy for cancer treatment. Our results show that high expression of Elovl6 in HCC is correlated with higher incidence of recurrence after tumor resection and poorer overall survival in patients with HCC. However, we were not able to identify a correlation between Elovl6 expression and the clinical parameters of disease severity. Liver tumor resection is not generally indicated in patients with advanced HCC. Of 61 cases, 50 (82%) cases were stage I, and 52 (85%) cases were moderately differentiated in our series. Therefore, Elovl6 may be an important prognostic factor in resected HCC independent of stage and differentiation. Further investigation is required to determine the expression of Elovl6 in advanced and unresectable HCC.

Fatty acid synthase facilitates lipogenesis and is significantly upregulated in many types of cancer^13^. In breast and pancreatic cancers, it has an active role in chemotherapy resistance. Several fatty acid synthase inhibitors, including cerulenin, C75, orlistat, C93, GSK837149A, and natural plant-derived polyphenols, have been shown to exert antitumor activities. A combination of cerulenin and trastuzumab synergistically downregulates ErbB2 expression, leading to more effective inhibition of tumor growth^14–16^. A novel Elovl6 inhibitor, 5,5-dimethyl-3-(5-methyl-3-oxo-2-phenyl-2,3-dihydro-1H-pyrazol-4-yl)-1-phenyl-3-(trifluoromethyl)-3,5,6,7-tetrahydro-1H-indole-2,4-dione, has been reported. This Elovl6 inhibitor was shown to display more than 30-fold greater selectivity for Elovl6 over other Elovl family members and effectively reduced the elongation index of fatty acids of hepatocytes^17^. However, the effect of this Elovl6 inhibitor on cellular proliferation remains unclear. We show that suppression of Elovl6 inhibits HCC cell proliferation *in vitro* and tumor growth *in vivo*. These findings highlight a potential strategy for cancer therapy in the future.

## Methods

### Human HCC tissues

The samples stained for Elovl6 were from a retrospective study including 61 patients who underwent liver resection for HCC between 2004 and 2009 at Chi Mei Medical Center (Tainan, Taiwan). For real-time RT-PCR analysis, 30 tumor and non-tumor HCC pairs were randomly selected from the Tumor Bank at Chi-Mei Medical Center. Pathological, demographic, and survival data of these patients were retrieved from medical records. All tumors were harvested from primary liver sites. All tumors were diagnosed using histology and graded according to Edmondson’s scales and classified as well differentiated, moderate, and poorly differentiated. The non-tumor samples were taken from gross normal liver tissue away from the tumor. This study was approved by the Institutional Review Board and Human Ethics Committee of Chi Mei Medical Center (IRB Serial No.:10212-001) and was performed in accordance with the approved guidelines. Informed consent was obtained from all participants. The evaluation of patients included medical records, physical examination, and a blood test. All patients received screen tests for hepatitis B surface antigen (HBsAg) and hepatitis C antibody (HCVAb). Subjects were classified as having diabetes mellitus or not based on past medical history, and measurement of body mass index was determined before the tumor tissue harvest.

### Immunohistochemistry staining

Sixty-one patients with HCC were used for detection of Elovl6 expression. An intensity score was assigned, which represented the average intensity of staining of the positive tumor cells, which were classified as negative, weak, moderate, and strong, as described previously^18^. The H-score was calculated as previously described^19^, where a score ≥ 140 was considered high expression.

### Oligonucleotides

The following oligonucleotides were used for the RT-PCR: Elovl6, 5′-TGCTCCTG-TACTCCTGGTACTCC-3′ (forward) and 5′-TTCTTCACTTTGCCGATGTAGG-3′ (reverse); SREBP-1c, 5′- GGACATCTTGCTGCTTCTAACCTGG-3′ (forward) and 5′-TGCCTCTTCATCCCGCCTCA-3′ (reverse); ACC, 5′-GCAGGTATCCCAACT-CTTCCC-3′ (forward) and 5′-TTCTGATCCCTTTCCCTCCTC-3′ (reverse); FAS, 5′-CGGGTCTATGCCACGATTCT-3′ (forward) and 5′-CACAGGGACCGAGTAA-TGCC-3′ (reverse); SCD-1, 5′-GATCATACTGGTTCCCTCCTGC-3′ (forward) and 5′-GTGGGCGTGTGTTTCTGAGA-3′ (reverse); CPT-1, 5′-GCTCGCACATTACA-AGGACATG-3′ (forward) and 5′-CTTGGACACCACATAGAGGCAG-3′ (reverse); GAPDH, 5′-ACTTCAACAGCGACACCCACT-3′ (forward) and 5′-GCCAAATTC-GTTGTCATACCAG-3′ (reverse); TATA-binding protein (TBP), 5′-TGTAAACTTGACCTAAAGACCATTGC-3′ (forward) and 5′-TGTTCTTCACTCT-TGGCTCCTGT-3′ (reverse). The relative expression levels were analyzed using the comparative Ct method and normalized to those of TBP or GAPDH in the clinical tissues and ML-1 cells.

### Antibodies

For immunoblotting, anti-PTEN antibody (9188), anti-phospho-Akt (Ser 473) antibody (9271), anti-Akt antibody (9272), and anti-ERK antibody (4695) were purchased from Cell Signaling Technology. Anti-Elovl6 antibody was purchased from Atlas Antibody AB (AlbaNova University Center, SWEDEN). Anti-phospho-ERK antibody (sc-7393), anti-p21 antibody (sc-56335), anti-cyclin D1 antibody (sc-753), anti-cyclin E antibody (sc-198), and anti-CDK4 antibody (sc-260) were purchased from Santa Cruz Biotechnology. Anti-β-actin-HRP antibody (A3854), palmitate (P0500), palmitoleate (P9417), stearate (S4751), and Oleate (O1383) were purchased from Sigma-Aldrich. For immunofluorescence, anti-Cx32 antibody was purchased from Abcam. Dylight™ 488-conjugated goat anti-rabbit IgG was purchased from Invitrogen. DAPI was purchased from Sigma-Aldrich. The fluorescence signals were detected with an Olympus FV1000 MPE Multiphoton Laser Scanning Microscope.

### Adenovirus production and titration

The adenoviral plasmid used for producing adenovirus Ad.shElovl6 was generously provided by N. Yamada^2^. The production and titration of adenoviruses were prepared as previously described^20^.

### BODIPY and PI staining

Adenovirus-infected ML-1 cells were fixed in 4% paraformaldehyde. After staining with BODIPY 505/515 reagent (Invitrogen), the cells were observed using fluorescence microscopy. For PI staining, the cells were mixed with 1 ml propidium iodide solution (200 μg/ml RNase, 20 μg/ml propidium iodide, and 0.1% Triton X-100) and incubated for 45 minutes. Flow cytometry was assessed using FACScan (BD Biosciences) and analyzed using WinMDI 2.9 software (Scripps Research Institute).

### Cell viability

Adenovirus-infected cells were incubated with fatty acids for 72 h, followed by treatment with WST-8 reagent (Dojindo Laboratories) for 1 h. The absorbance at 450 nm was measured, and the percentage of cell viability was determined by the ratio of O.D. values measured at each fatty acid concentration to that of 0 μM.

### Animal experiments

Six to eight-week-old male BALB/c mice were purchased from the Animal Center at National Cheng Kung University Medical College. All experimental procedures were approved by the Laboratory Animal Care and Use Committee of National Cheng Kung University and performed in accordance with the approved guidelines. ML-1 cells (10^6^) were subcutaneously injected into the BALB/c mice. For virus treatment, 10^8^ plaque forming units (PFU) of Ad.shElovl6 were injected intratumorally on days ten and sixteen. Tumor volumes were determined as previously described^19^.

### Statistical analysis

Differences in tumor volume between the two groups were compared by repeat-measures analysis of variance (ANOVA) using SAS software (Strategic Application System). The survival analysis was determined using the Kaplan-Meier survival curve and the log-rank test.

## Electronic supplementary material

Supplementary information
