# Supplementary material for: Elovl6 is a negative clinical predictor for liver cancer and knockdown of Elovl6 reduces murine liver cancer progression
Source: Sci Rep. 2018 Apr 26;8:6586. doi: 10.1038/s41598-018-24633-3 (PMC5920119; doi:10.1038/s41598-018-24633-3)
Supplement: Supplementary file 1 — Supplementary information [file 41598_2018_24633_MOESM1_ESM.docx]

**Elovl6 is a negative clinical predictor for liver cancer and knockdown of Elovl6 reduces murine liver cancer progression**

Yu-Chu Su, Yin-Hsun Feng, Hung-Tsung Wu, Yao-Shen Huang, Chao-Ling Tung, Pensee Wu, Chih-Jen Chang, Ai-Li Shiau, and Chao-Liang Wu

**Supplemental Data**

**Supplemental Figure S1**

**Figure S1.** Detection of apoptotic cells using TUNEL assay in Elovl6 knockdown ML-1 cells. Scale bar = 200 μm; original magnification, 200×.

**Supplemental Figure S2**

**Figure S2.** Expression of cleaved caspase-3 was examined in Elovl6 knockdown tumor samples. Scale bar = 50 μm; original magnification, 200×.

**Supplemental Figure S3**

**Figure S3.** The full-length blots of each cropped western blotting in Figure 4.
